# Distinctive Regulation of Carbapenem Susceptibility in *Pseudomonas aeruginosa* by Hfq

**DOI:** 10.3389/fmicb.2020.01001

**Published:** 2020-05-26

**Authors:** Elisabeth Sonnleitner, Petra Pusic, Michael T. Wolfinger, Udo Bläsi

**Affiliations:** ^1^Department of Microbiology, Immunobiology and Genetics, Max Perutz Labs, Vienna BioCenter (VBC), University of Vienna, Vienna, Austria; ^2^Department of Theoretical Chemistry, Faculty of Chemistry, University of Vienna, Vienna, Austria; ^3^Research Group Bioinformatics and Computational Biology, Faculty of Computer Science, University of Vienna, Vienna, Austria

**Keywords:** *Pseudomonas aeruginosa*, *opdP*, *oprD*, carbapenem resistance, riboregulation, catabolite repression

## Abstract

Carbapenems are often the antibiotics of choice to combat life threatening infections caused by the opportunistic human pathogen *Pseudomonas aeruginosa*. The outer membrane porins OprD and OpdP serve as entry ports for carbapenems. Here, we report that the RNA chaperone Hfq governs post-transcriptional regulation of the *oprD* and *opdP* genes in a distinctive manner. Hfq together with the recently described small regulatory RNAs (sRNAs) ErsA and Sr0161 is shown to mediate translational repression of *oprD*, whereas *opdP* appears not to be regulated by sRNAs. At variance, our data indicate that *opdP* is translationally repressed by a regulatory complex consisting of Hfq and the catabolite repression protein Crc, an assembly known to be key to carbon catabolite repression in *P. aeruginosa*. The regulatory RNA CrcZ, which is up-regulated during growth of *P. aeruginosa* on less preferred carbon sources, is known to sequester Hfq, which relieves Hfq-mediated translational repression of genes. The differential carbapenem susceptibility during growth on different carbon sources can thus be understood in light of Hfq-dependent *oprD*/*opdP* regulation and of the antagonizing function of the CrcZ RNA on Hfq regulatory complexes.

## Introduction

The opportunistic human pathogen *Pseudomonas aeruginosa* can cause severe infections. It is particularly devastating for immunocompromised individuals and patients with cystic fibrosis, leading to high morbidity and mortality. As they are less prone to degradation by extended spectrum β-lactamases, carbapenems are frequently used to treat severe infections of Gram-negative bacteria including *P. aeruginosa* (Papp-Wallace et al., 2011; Fritzenwanker et al., 2018). Carbapenem-resistant *P. aeruginosa* strains are increasingly occurring (McDougall et al., 2013; Castanheira et al., 2014; Buehrle et al., 2017), which prompted the World Health Organization to rank carbapenem-resistant *P. aeruginosa* among the priority pathogens to investigate new drug treatments (Tacconelli et al., 2018). *P. aeruginosa* exhibits several resistance mechanisms toward carbapenems including the production of metallo-β-lactamases and carbapenemase (Sacha et al., 2008; Bassetti et al., 2018) as well as dedicated efflux systems (Chalhoub et al., 2016). Another notable feature of *P. aeruginosa’s* high intrinsic antibiotic resistance is the low outer membrane permeability (Hancock and Woodruff, 1988; Breidenstein et al., 2011). *P. aeruginosa* utilizes a variety of specialized outer membrane porins (Hancock and Brinkman, 2002; Eren et al., 2012). Two of them, OprD/OccD1 and OpdP/OccD3, show high sequence similarity (51%) and serve as entry ports for basic amino acids and small peptides as well as for certain carbapenems, e.g., imipenem and meropenem (Quinn et al., 1986; Trias et al., 1989; Tamber and Hancock, 2006; Papp-Wallace et al., 2011; Isabella et al., 2015). In fact, the first documented case of clinical resistance to carbapenems was found to be due to a loss of the monocistronic *oprD* gene (Quinn et al., 1986). The deletion of *oprD* resulted in a decreased susceptibility to carbapenems, but deletion of the *opdP* gene alone, residing in an operon together with genes encoding a dipeptide ABC transport system (Chevalier et al., 2017), did not cause a significant change. However, the deletion of both genes led to a remarkable increase in resistance when compared to the deletion of *oprD* alone (Isabella et al., 2015).

The RNA chaperone Hfq is a pleiotropic regulator and virulence factor in *P. aeruginosa* (Sonnleitner et al., 2003, 2006, 2018). Hfq is involved in the control of mRNA translation through distinct mechanisms. In riboregulation, Hfq can act indirectly by facilitating base-pairing interactions of small regulatory RNAs (sRNAs) with cognate mRNA targets (Vogel and Luisi, 2011; Kavita et al., 2018). On the other hand, Hfq can directly repress translation, by binding to A-rich sequences at or in the vicinity of translation initiation sites (Sonnleitner and Bläsi, 2014). Hfq has several distinct RNA binding sites. Crystallographic and biophysical data showed that RNA recognition is mediated by distinct interactions with distal, proximal, and rim faces of the hexameric ring (Schumacher et al., 2002; Link et al., 2009; Sauer et al., 2012; Panja et al., 2013). Many sRNAs bind to the proximal side of Hfq *via* U-rich stretches (Schumacher et al., 2002; Mikulecky et al., 2004; Link et al., 2009) or through the poly-uridine tails derived from rho-independent terminators (Otaka et al., 2011; Sauer and Weichenrieder, 2011; Ishikawa et al., 2012). Internal U/A-rich regions in sRNAs as well as in mRNAs were found to interact with arginine patches on the lateral rim of the Hfq-hexamer (Sauer et al., 2012; Peng et al., 2014; Schu et al., 2015). The distal side recognizes A-rich regions, previously defined as ARN repeats, where A is an adenine, R is any purine nucleotide and N is any nucleotide, which are often present in mRNAs around the ribosome binding site (Link et al., 2009; Murina et al., 2013; Robinson et al., 2014; Sonnleitner and Bläsi, 2014).

A GRIL-seq approach identified two base-pairing small regulatory sRNAs, Sr0161, and EsrA, as negative translational regulators of *oprD* (Zhang et al., 2017). ErsA is transcriptionally controlled by the envelope stress response regulator AlgU/T (Ferrara et al., 2015), and its expression is further up-regulated after a shift from high to low oxygen supply, and upon entry into stationary phase (Ferrara et al., 2015; Zhang et al., 2017). Sr0161 did not show any phase dependent expression in full broth (Zhang et al., 2017). The study by Zhang et al. (2017) also suggested an interaction between Sr0161 and *opdP* mRNA. RT-qPCR showed that the *opdP* levels were decreased after overexpression of the sRNA and modestly increased in a *sr0161* deletion mutant (Zhang et al., 2017). However, in contrast to Hfq-Sr0161/EsrA-mediated negative translational regulation of *oprD*, it remains unknown whether Sr0161 directly regulates translation of *opdP* (Zhang et al., 2017).

In *P. aeruginosa*, carbon catabolite repression (CCR) operates at the post-transcriptional level. In the presence of preferred C-sources (e.g., succinate), the RNA chaperone Hfq binds to the translation initiation region (TIR) of mRNAs encoding proteins important for the uptake and utilization of less preferred C-sources, and represses their translation (Sonnleitner and Bläsi, 2014). Upon binding, the Hfq/RNA complex forms a “platform” to which the catabolite repression protein Crc can bind, which results in stabilization of the repressive complex (Sonnleitner et al., 2018; Pei et al., 2019). When the preferred C-source is exhausted, the levels of the Hfq-titrating RNA CrcZ increase, which in turn leads to relieve of Hfq/Crc-mediated CCR (Sonnleitner and Bläsi, 2014). Previous studies provided some hints that *oprD* is regulated by CCR (Ochs et al., 1999; Linares et al., 2010). However, recent omics studies are somewhat inconsistent. A ChIP-seq approach identified *oprD* mRNA among the mRNAs that were co-immunoprecipitated with antibodies directed against Hfq as well as against Crc (Kambara et al., 2018). Nevertheless, transcriptomic and proteomic analyses designed to reveal the Crc regulon revealed only *opdP* as a candidate for Crc-mediated post-transcriptional regulation but not *oprD* (Corona et al., 2018).

Taken these studies together with the observation that a PAO1*hfq*-deletion mutant was more susceptible to carbapenems when compared with the parental strain (Ducret et al., 2016; Pusic et al., 2018), it appeared safe to assume that Hfq is involved in regulation of both, *oprD* and *opdP*. In this study, we addressed the question whether post-transcriptional regulation of *oprD* and *opdP* occurs through riboregulation and/or through CCR, i.e., through Hfq/Crc repressive complexes. To dissect the regulatory mechanism(s) for *oprD* and *opdP*, we used a genetic approach employing translational reporter constructs in conjunction with different *P. aeruginosa* O1 mutant strains. These studies verify that *oprD* is regulated by Hfq-mediated riboregulation and strongly indicate that *opdP* is directly translationally repressed by Hfq/Crc.

## Materials and Methods

### Bacterial Strains, Plasmids, and Growth Conditions

Strains and plasmids used in this study are listed in Supplementary Table S1. If not indicated otherwise, the cultures were grown at 37°C in either Lysogeny-broth (LB) (Sambrook et al., 1989) or basal-salt medium (BSM) supplemented with the indicated carbon sources (Sonnleitner et al., 2009). When required, the following concentrations of antibiotics were used: 15 μg/ml gentamicin, 25 μg/ml tetracycline, and 100 μg/ml ampicillin for *Escherichia coli*; 50 μg/ml gentamicin, 100 μg/ml of tetracycline, and 250 μg/ml carbenicillin for *P. aeruginosa*.

### Construction of PAO1 Deletion Strains

The deletion of the first 82 nucleotides (nt) of *ersA* (coordinates 6183580-6183661 of the PAO1 genome; Winsor et al., 2016) and the deletion of the *sr0161* 3′ end including additional 24 nt downstream of *sr0161* (coordinates 184302–184482 of the PAO1 genome; Winsor et al., 2016) was created by homologous recombination, respectively (Zhang et al., 2017). Briefly, plasmids pEXG2-*sr0161* (for generation of PAO1Δ*sr0161* and PAO1Δ*hfq*Δ*sr0161*) and pEXG2-*ersA* (for generation of PAO1Δ*ersA*Δ*sr0161* and PAO1Δ*hfq*Δ*ersA*Δ*sr0161*) were mobilized into the strains PAO1, PAO1Δ*hfq*, PAO1Δ*sr0161*, and PAO1Δ*hfq*Δ*sr0161* with the aid of *E. coli* strain S17-1, and then chromosomally integrated through selection for gentamicin. Excision of the vector by a second crossover event was achieved by selection of sucrose insensitive cells as the pEXG2 vector encodes the *Bacillus subtilis sacB* gene, whose gene product – levan sucrase – renders Gram-negative cells sensitive to sucrose (Rietsch et al., 2005; Hmelo et al., 2015).

### Construction of Plasmid pME6015P_tac_

For construction of a cloning vector suited for the generation of translational *lacZ* fusions transcribed under the control of the P_tac_ promoter, a 1,522-base pair (bp) fragment encoding the LacI^q^ repressor gene and the P_tac_ promotor together with the operator sites located downstream of the transcriptional start site were amplified by PCR using the oligonucleotides L85 (5′-GAT ATC **GAA TTC** GAA CGC CAG CAA GAC-3′) and M85 (5′-TTT TT**G GAT CC** A ATT GTT ATC CGC TCA CAA TTC C-3′) and plasmid pME6032 as template. The PCR fragment was cleaved with *Eco*RI and *Bam*HI, and then ligated into the corresponding sites of plasmid pME6015.

### Construction of Plasmids pME6015P_tac_*oprD*::*lacZ* and pME6015P_tac_*opdP::lacZ*

To construct translational gene fusions between *oprD* and *opdP*, respectively, and *lacZ* under transcriptional control of the P_tac_ promoter, a 71-bp fragment of the TIR of *oprD* (coordinates 1045365–1045294 of the PAO1 genome; Winsor et al., 2016) and a 118-bp fragment of the TIR of *opdP* (coordinates 5038800–5038918 of the PAO1 genome; Winsor et al., 2016) were amplified by PCR using the oligonucleotide pairs A123 (5′-ACG T**GG ATC C** AC AAG AAG AAC TAG CCG TCA C-3′)/H112 (5′-ACG T**CT GCA G** GC TCC ACT TCA TCA CTT TCA TTG-3′) for *oprD* and Q144 (5′-ACG TGG ATC CTC GCC GCG CCG TCT TCG-3′)/O144 (5′-ACG T**CT GCA G** AG CGT TCC TGG CGG AAC-3′) for *opdP*, respectively, and chromosomal DNA of PAO1 as template. The PCR fragments were cleaved with *Bam*HI and *Pst*I, and then ligated into the corresponding sites of plasmid pME6015P_tac_. Despite the presence of LacI^q^, the P_tac_ promoter was leaky in the pME6015P_tac_ derivatives (not shown). Thus, IPTG was not added in the experiments described below.

### Co-immunoprecipitation of mRNAs Bound to Hfq

A total of 40 ml of PAO1 culture was grown in BSM complex medium (Sonnleitner et al., 2018) and harvested at an OD_600_ of 1.5. The cells were first washed in lysis buffer (20 mM Tris pH 8.0, 150 mM KCl, 1 mM MgCl_2_, 1 mM DTT, and 0.05% Triton X-100) and then snap frozen in liquid nitrogen. The cells were lysed by sonication (six times for 10 sec on ice) in 800 μl lysis buffer in the presence of 200 U RiboLock^®^ RNase inhibitor (Fermentas). Cell debris were removed by centrifugation and anti-Hfq antibodies (Pineda) were added to 60 μl supernatant and incubated for 2 h at 4°C on a rotating wheel. Then, 5 μl Dynabeads^®^ Protein G beads (Novex) were added, and the incubation was continued for 1 h. The beads were washed three times with lysis buffer and finally collected in 200 μl of lysis buffer without Triton X-100. Then, 100 μl phenol was added and the beads were shaken at 900 rpm for 30 min at room temperature. The RNA was purified by phenol-chloroform extraction, which was followed by ethanol precipitation. Libraries were constructed using NEBNext^®^ Ultra^TM^ Directional RNA Library Prep Kit from Illumina. A total of 100 bp single end sequence reads were generated using the Illumina HiSeq 2000 platform at the Vienna BioCenter Core Facility^1^. Sequencing adapter removal was performed with cutadapt (Martin, 2011). Mapping of *oprD* and *opdP* RNA against the PAO1 reference genome (NCBI accession number NC_002516.2) was performed with Segemehl (Hoffmann et al., 2009) with default parameters. The mapped sequencing data were prepared for visualization using the ViennaNGS tool box, and visualized with the UCSC Genome Browser (Wolfinger et al., 2015). The raw sequencing data were deposited in the European nucleotide archive (ENA) as a study under the accession number PRJEB37368.

### Microscale Thermophoresis

For *in vitro* transcription of the 100 nt long *oprD* RNA fragment [nt −61 to nt +39 with regard to the A (+1) of the AUG start codon] and the 150 nt long *opdP* RNA fragment [nt −111 to nt +39, with regard to the G (+1) of the GUG start codon], the AmpliScribe T7-Flash Transcription Kit (Epicentre Biotechnologies) was used according to the manufacturer’s instructions. First, PCR fragments were generated with the primer pairs Y133 (5′-TCT AGA CGT AAT ACG ACT CAC TAT AGG CTA GCC GTC ACT GCG GCA C-3′)/Z133 (5′-AAC CGC CAG TGC AAT GGC-3′) (*oprD*) and B148 (5′-TCT AGA CGT AAT ACG ACT CAC TAT AGG CCA GGA GCG CTC GCC TC-3′)/C148 (5′-TCC CAG CGT CGC GCC GGT C-3′) (*opdP*), and *P. aeruginosa* chromosomal DNA. The forward primers contained a T7 promoter sequence (underlined).

For microscale thermophoresis (MST) (Wienken et al., 2010), 20 μM of Hfq protein was labeled with Monolith NT^TM^ Protein Labelling Kit RED-NHS according to the manufacturer’s instructions (Nanotemper Technologies). For determination of protein-RNA interactions, 56 nM labeled Hfq was used in the presence of increasing concentrations of non-labeled *in vitro* transcribed RNA (either *oprD*_–__61 –+ 39_ or *opdP*_–__111 –+ 39_). The ligands were dissolved in MST-buffer (50 mM Tris pH 7.4, 150 mM NaCl, and 10 mM MgCl_2_) in the presence of 0.1% Tween-20 and 0.1% BSA. After 30 min incubation at 37°C, the samples were loaded onto NT.115 hydrophilic capillaries (Nanotemper Technologies) and measured in an MST Monolith NT.115 Green/Red instrument at the Vienna BioCenter Core Facility^2^. The MST measurements were performed in duplicate. The data for MST analysis were recorded at 25°C using the red LED (excitation: 625 nm and emission: 680 nm); MST Power 40%, LED Power 50%. Data analysis was performed with NTAffinityAnalysis v2.0.2 for thermophoresis and T-jump analysis 0 and 5 s after the pulse. For determination of the *K*_*d*_-values the Hill-fit model within the NTAffinityAnalysis software was used.

### Agar Disk Diffusion Assay

PAO1 and mutants thereof were grown in BSM supplemented with either 40 mM succinate or mannitol to an OD_600_ of 1.8–2.0. Then, 200 μl of cultures were plated on agar plates containing the respective media and filter disks loaded with 10 μg imipenem were applied on top (Oxoid). The plates were incubated at 37°C. The diameter of the growth inhibition zones was measured in mm.

### β-Galactosidase Assays

The β-galactosidase activities were determined as described (Miller, 1972). The cells were permeabilized with 5% toluene. Unless indicated otherwise, the β-galactosidase units in the different experiments were derived from at least two independent experiments, and are shown as mean. The error bars in the different figures represent standard deviations. Except for the data in Figure 3, statistical analyses were performed in Excel with a two tailed distributed Student’s *T*-test of two sample arrays with unequal variance, ns (non-significant); *P* > 0.05, ^∗^*P* ≤ 0.05, ^∗∗^*P* ≤ 0.01, and ^∗∗∗^*P* ≤ 0.001. Due to the multiple comparison in Figure 3, the results were statistically analyzed by an ANOVA test with *post hoc* multiple comparison. In short, Levene’s test was used to test for equality (homogeneity) of variances between the tested groups. Statistical significance was determined by one-way ANOVA with the Tukey’s *post hoc* test when more than two groups with normal distribution were compared (Figure 3A). When more than two groups with unequal variance were compared, the Brown-Forsythe and Welch’s ANOVA test with the Dunnett’s T3 *post hoc* test was used (Figures 3B–D), ns (non-significant); ^∗^*P* < 0.05, ^∗∗^*P* < 0.01, and ^∗∗∗^*P* < 0.001 were considered statistically significant results.

### Western-Blot Analyses

Equal amounts of total proteins were separated on a 12% SDS-polyacrylamide gel, and then electro-blotted onto a nitrocellulose membrane. The blots were blocked with 5% dry milk in TBS buffer, and probed with rabbit anti-Hfq, rabbit anti-Crc or rabbit anti-*E. coli*-S1 antibodies (Sonnleitner and Bläsi, 2014). Immunodetection of ribosomal protein S1 served as a loading control. The antibody–antigen complexes were visualized with horseradish peroxidase (HRP) conjugated anti-rabbit antibodies (Cell Signaling Technology) using the SuperSignal^TM^ West Pico PLUS chemiluminescent substrate kit (Thermo Scientific). The signals were detected with ChemiDoc^TM^ Touch Imaging System (BioRad) and analyzed with ImageLab 5.2.1 (BioRad).

## Results

### Hfq Is Involved in Translational Regulation of *oprD* and *opdP*

**FIGURE 1:**
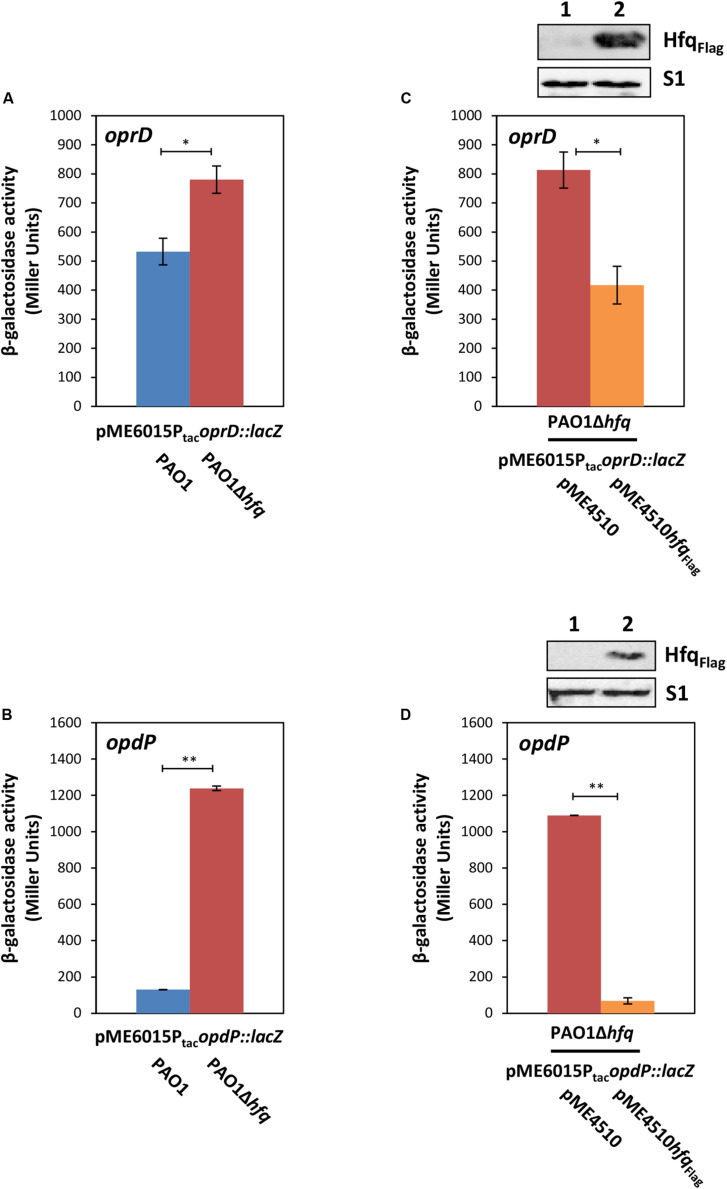
Hfq negatively regulates *oprD* and *opdP* translation. **(A,B)** The strains PAO1 (blue bar) and PAO1Δ*hfq* (red bar) harboring either plasmid pME6015P_tac_*oprD::lacZ* **(A)** or pME6015P_tac_*opdP::lacZ* **(B)** were grown in LB medium. Samples were withdrawn at an OD_600_ of 2.0. The bars represent the β-galactosidase values conferred by the *oprD::lacZ* translational fusion gene encoded by plasmid pME6015P_tac_*oprD::lacZ* **(A)** and by the *opdP::lacZ* translational fusion gene encoded by plasmid pME6015P_tac_*opdP::lacZ* **(B)**, respectively. The error bars represent standard deviations from two independent experiments. The strains PAO1Δ*hfq*(pME4510) (red bar) and PAO1Δ*hfq*(pME4510*hfq*_Flag_) (orange bar) harboring either plasmid pME6015P_tac_*oprD::lacZ* **(C)** or pME6015P_tac_ *opdP::lacZ* **(D)** were grown in LB medium. Samples were withdrawn at an OD_600_ of 2.0. The bars represent the β-galactosidase values conferred by the *oprD::lacZ* translational fusion gene **(C)** and by the *opdP::lacZ* translational fusion gene **(D)**, respectively, in the presence or absence of ectopic *hfq*_Flag_ expression. The error bars represent standard deviations from two independent experiments. Statistical analyses were performed in Excel with a two tailed distributed Student’s *T*-test of two sample arrays with unequal variance. ns *P* > 0.05, *P ≤ 0.05, and **P ≤ 0.01. (**C,D:** top panels), Hfq and S1 levels in strains PAO1Δ*hfq*(pME4510) (lane 1) and PAO1Δ*hfq*(pME4510*hfq*_Flag_) (lane 2) harboring either plasmid pME6015P_tac_*oprD::lacZ* **(C)** or pME6015P_tac_*opdP::lacZ* **(D)**. The Hfq levels were determined by Western-blot analyses using anti-Hfq antibodies. Immunodetection of ribosomal protein S1 served as a loading control.

Recent studies revealed that *P. aeruginosa hfq* deletion mutants showed an increased susceptibility to imipenem (Ducret et al., 2016; Pusic et al., 2018), which coincided with higher *oprD* and *opdP* transcript levels in the *hfq* mutant strain when compared with the parental strain during growth in different media (Supplementary Figure S1 and Supplementary Table S2) (Ducret et al., 2016; Pusic et al., 2018). To verify the impact of Hfq on *oprD* and *opdP* translation, *oprD::lacZ* and *opdP*::*lacZ* translational gene fusions were constructed and the β-galactosidase activities conferred by the respective fusion proteins were determined in strains PAO1 and PAO1Δ*hfq* harboring plasmids pME6015P_tac_*oprD::lacZ* and pME6015P_tac_*opdP::lacZ*, respectively. Transcription of either chimeric gene was driven by the P_tac_ promoter to control for potential transcriptional effects of Hfq (Ducret et al., 2016). The strains were grown in LB medium to an OD_600_ of 2.0, as the sRNAs ErsA and Sr0161 are transcribed under these conditions (Zhang et al., 2017). As reported by Zhang et al. (2017), our RNAseq analysis confirmed growth independent quantities of Sr0161 RNA in LB medium and increased levels of ErsA RNA in stationary phase when compared to logarithmically growing cells (Supplementary Figure S2). Translation of both reporter fusions was repressed in the presence of Hfq. However, the effect of Hfq on *oprD::lacZ* translation was less pronounced (Figure 1A; 1.5-fold difference between PAO1 and PAO1Δ*hfq* harboring pME1615P_tac_*oprD::lacZ*) than on *opdP::lacZ* translation (Figure 1B; 10-fold difference between PAO1 and PAO1Δ*hfq* harboring pME1615P_tac_*opdP::lacZ*). The differential translation rates observed for *oprD::lacZ* and *opdP::lacZ* in the wild-type strain and the corresponding *hfq* deletion strains coincided with a ∼2–3-fold and ≥10-fold higher transcript abundance of *oprD* and *opdP*, respectively, in PAO1 and PAO1*hfq* during cultivation in different media (Supplementary Table S2). As anticipated, the complementation of *hfq* by ectopic expression of a plasmid borne *hfq*_Flag_ allele in strain PAO1Δ*hfq* resulted again in repression of the *oprD::lacZ* and *opdP::lacZ* reporter genes (Figures 1C,D), respectively.

As the mRNA sequences of the TIRs of the *oprD* and *opdP* genes in the clinical isolate PA14 showed only minor differences to PAO1 (Winsor et al., 2016), the same experiments were performed with the clinical isolate PA14 and the corresponding PA14Δ*hfq* mutant harboring plasmids pME1615P_ta__c_*oprD::lacZ* and pME1615P_tac_*opdP::lacZ*, respectively, after growth in synthetic cystic fibrosis medium (SCFM) containing 100 μM FeSO_4_ (Palmer et al., 2007; Tata et al., 2016). The outcome of these experiments was comparable with the results obtained for PAO1 after growth in LB medium (Supplementary Figures S3A–D).

### Binding of Hfq to the Translation Initiation Regions of *oprD* and *opdP*

**FIGURE 2:**
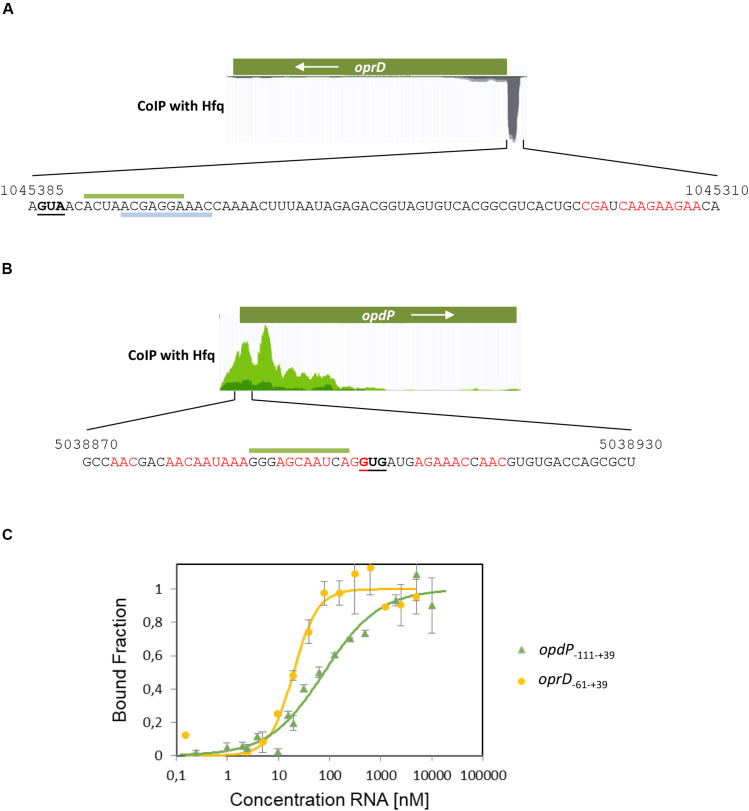
Co-immunoprecipitation of *oprD* and *opdP* mRNA fragments with Hfq. Hfq-bound RNAs were isolated from lysates of the PAO1 culture after co-immunoprecipitation with Hfq-specific antibodies as described in section “Materials and Methods.” The identity of Hfq-bound RNAs in the supernatant was revealed by RNA-seq. **(A,B)** The read coverage visualized in the Genome Browser is shown for *oprD* **(A)** and *opdP* **(B)**, respectively. The sequences of the co-immunoprecipitated mRNA fragments corresponding to the TIR of *oprD* **(A)** and *opdP* **(B)**, respectively, are shown below. The numbers refer to the PAO1 genome coordinates (http://www.pseudomonas.com) (Winsor et al., 2016). The start codons of *oprD* and *opdP* are underlined. Putative Hfq binding sites are indicated in red. The interacting sequences for EsrA and Sr0161 in the *oprD* TIR **(A)** (Zhang et al., 2017) are indicated by gray and green bars, respectively. The putative interacting sequence for Sr0161 in the *opdP* TIR **(B)** is indicated by a green bar **(B)** (Zhang et al., 2017). **(C)** Microscale thermophoresis reveals the *K*_*d*_ of Hfq for *oprD*_–__61 –+ 39_ RNA (yellow circles) and *opdP*_–__111 –+ 39_ RNA (green triangles) with 19.7 ± 2.8 nM and 78.1 ± 1.97 nM, respectively. Increasing amounts of the non-labeled *in vitro* transcribed *oprD*_–__61 –+ 39_ and *opdP*_–__111 –+ 39_ fragments were added to 56 nM fluorescently labeled Hfq protein. The dissociation constant (*K*_*d*_) of *oprD*_–__61 –+ 39_ and *opdP*_–__111 –+ 39_ was determined as described in section “Materials and Methods,” and was expressed as mean EC50 ± EC50 confidence interval of two independent experiments. Thermophoresis/T-jump analysis is shown. LED power of 50% and MST power of 40% were used.

To test whether Hfq binds to the TIRs of *oprD* and *opdP*, a co-immunoprecipitation assay with Hfq specific antibodies was performed to identify protein bound RNA fragments. Subsequent RNA-seq revealed a distinct sub-sequence upstream of the *oprD* start codon (Figure 2A). This region contains several ARN motifs, which have been shown to bind to the distal side of Hfq (Link et al., 2009). The sub-sequence also comprises the ErsA and Sr0161 interaction sites (Figure 2A) (Zhang et al., 2017).

**FIGURE 3:**
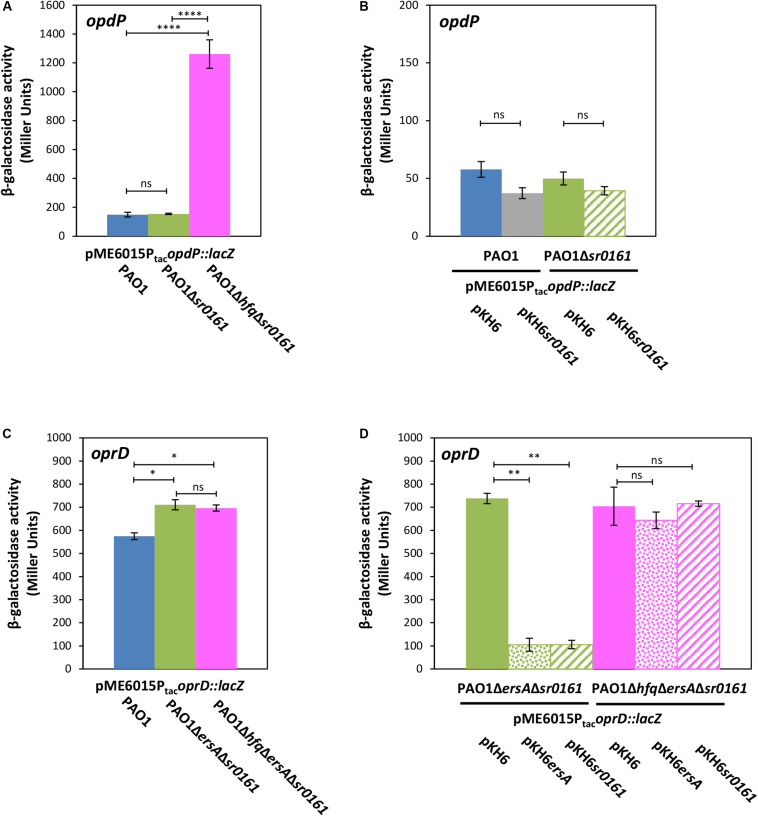
The sRNAs Sr0161 does not regulate *opdP* translation. **(A)** The strains PAO1 (blue bar), PAO1Δ*sr0161* (green bar), and PAO1Δ*hfq*Δ*sr0161* (pink bar) harboring plasmid pME6015P_tac_*opdP::lacZ* were grown in LB medium. Samples were withdrawn at an OD_600_ of 2.0. The bars represent the β-galactosidase values conferred by the *opdP::lacZ* translational fusion gene encoded by plasmid pME6015P_tac_*opdP::lacZ.* **(B)** The strains PAO1 (pKH6) (blue bar), PAO1(pKH6*sr0161*) (gray bar), PAO1Δ*sr0161*(pKH6) (green bar), and PAO1Δ*sr0161*(pKH6*sr0161*) (striped green bar) harboring plasmid pME6015P_tac_*opdP::lacZ* were grown in LB medium supplemented with 0.2% arabinose to induce sRNA gene expression from plasmid pKH6*sr0161*. Samples were withdrawn at an OD_600_ of 2.0. The bars represent the β-galactosidase values conferred by the *opdP::lacZ* translational gene fusion encoded by plasmid pME6015P_tac_*opdP::lacZ*. **(C)** The strains PAO1 (blue bar), PAO1Δ*ersA*Δ*sr0161* (green bar) and PAO1Δ*hfq*Δ*ersA*Δ*sr0161* (pink bar) harboring plasmid pME6015P_tac_*oprD::lacZ* were grown in LB medium. Samples were withdrawn at an OD_600_ of 2.0. The bars represent the β-galactosidase values conferred by the *oprD::lacZ* translational fusion gene encoded by plasmid pME6015P_tac_*oprD::lacZ*. **(D)** Ectopic expression of *ersA* and *sr0161* in strains PAO1Δ*ersA*Δ*sr0161* (green bars) and PAO1Δ*hfq*Δ*ersA*Δ*sr0161* (pink bars) harboring plasmid pME6015P_tac_*oprD::lacZ*. The β-galactosidase values conferred by the *oprD::lacZ* translational fusion gene in either strain in the absence of *ersA*/*sr0161* expression (solid bars) and in the presence of *ersA* (dotted bars) or *sr0161* (striped bars) are indicated by bars. Statistical analyses were performed with AGOVA test with *post hoc* multiple comparison as described in section “Materials and Methods.” ns (non-significant); *P < 0.05, **P < 0.01, ***P < 0.001, ****P < 0.0001.

The co-immunoprecipitated RNA sub-sequence including the start codon of *opdP* comprised as well several ARN-triplets and overlaps with the predicted Sr0161 binding-site (Figure 2B) (Zhang et al., 2017).

The binding affinity of Hfq for the respective co-immunoprecipitated *oprD* and *opdP* sub-sequences was determined by MST. Hfq exerted a high affinity for the *oprD*_–__61 –+ 39_ sub-sequence (*K*_*d*_ = 19.7 ± 2.8 nM) and a somewhat lower affinity for the *opdP*_–__111 –+ 39_ sub-sequence (*K*_*d*_ = 78.1 ± 1.97 nM) (Figure 2C).

At this junction it seems worth noting that binding of Hfq to the TIRs of either *oprD* or *opdP* is anticipated regardless of whether Hfq/sRNA-mediated regulation is the underlying mechanism of translational repression, or whether this occurs through Hfq/Crc repressive complexes.

### The sRNA Sr0161 Does Not Impact Translation of *opdP*

Next, we re-examined whether sRNA Sr0161, which was identified by GRIL-seq and suggested to base-pair with the *opdP* TIR (Zhang et al., 2017) can translationally regulate *opdP*. The strains PAO1 and PAO1Δ*sr0161* harboring plasmid pME6016P_tac_*opdP::lacZ* were grown in LB medium to an OD_600_ of 2.0. The β-galactosidase activities conferred by the translational *opdP::lacZ* reporter gene were indistinguishable in either strain (Figure 3A). In addition, ectopic expression of Sr0161 in PAO1 and PAO1Δ*sr0161* harboring plasmid pME6016P_tac_*opdP::lacZ* did not result in significant translational repression of the *opdP::lacZ* reporter gene (Figure 3B), arguing against translational regulation of *opdP* by Sr0161. However, the β-galactosidase activity conferred by the *opdP::lacZ* reporter gene encoded by plasmid pME6015P_tac_*opdP::lacZ* was approximately 10-times higher in strain PAO1Δ*hfq*Δ*sr0161* when compared with strains PAO1 and PAO1Δ*sr0161* (Figure 3A), suggesting that Hfq regulates *opdP* either with the aid of an hitherto unknown sRNA or directly by a Hfq/Crc repressive complex.

On the other hand, our studies with the constructed PAO1Δ*ersA*Δ*sr0161*(pME6015P_tac_*oprD::lacZ*) double mutant and with the PAO1*hfq*Δ*ersA*Δ*sr0161*(pME6015P_tac_*oprD::lacZ*) triple mutant strain showed that the absence of the sRNAs leads to de-repression of *oprD::lacZ* translation in both strains (Figure 3C), whereas ectopic overexpression of either sRNA restored translational repression of *oprD::lacZ* in strain PAO1Δ*ersA*Δ*sr0161*(pME6015P_tac_*oprD::lacZ*) to the same level but not in strain PAO1Δ*hfq*Δ*ersA*Δ*sr0161* (pME6015P_tac_*oprD::lacZ*) (Figure 3D). Thus, the latter studies corroborate and extend the observations of Zhang et al. (2017) in that (i) EsrA and Sr0161 negatively regulate *oprD* translation and that (ii) not only Sr0161 operates in a Hfq-dependent manner but also EsrA.

### Crc Impacts Hfq-Mediated *opdP* Regulation but Not on Hfq/EsrA/Sr0161-Mediated Riboregulation of *oprD*

**FIGURE 4:**
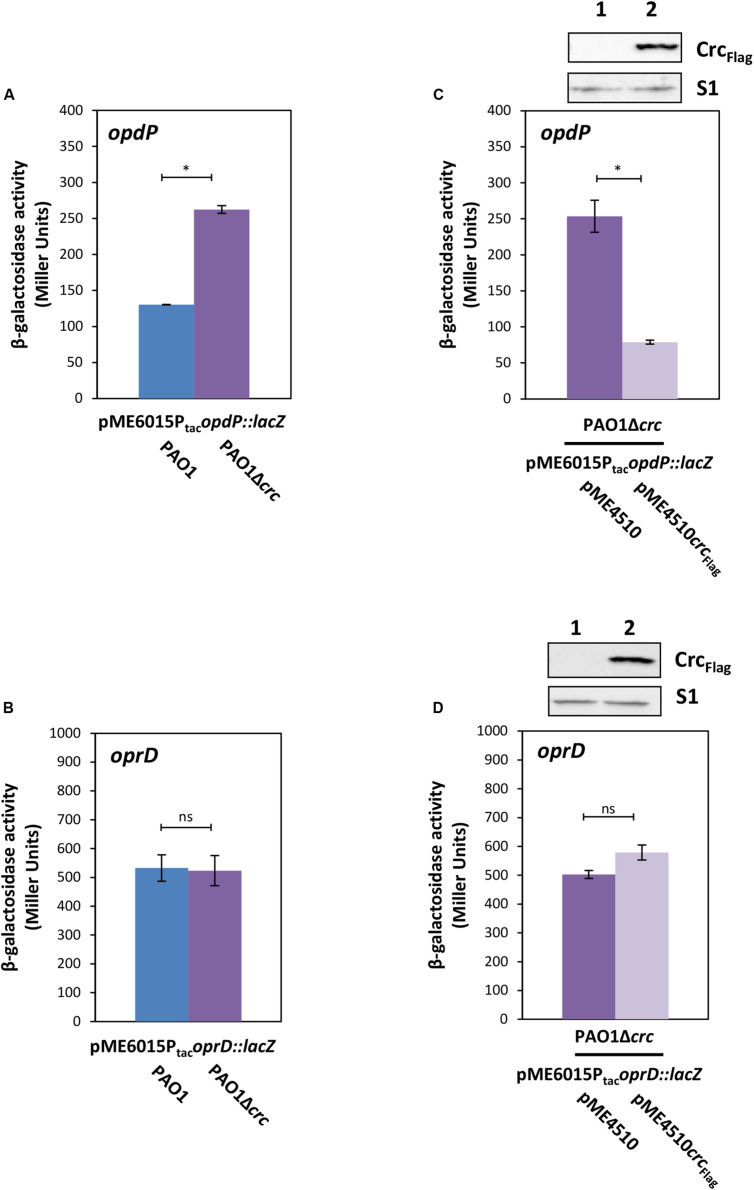
Crc impacts on *opdP* but not on *oprD* translation. **(A,B)** The strains PAO1 (blue bar) and PAO1Δ*crc* (purple bar) harboring either plasmid pME6015P_tac_*opdP::lacZ* **(A)** or pME6015P_tac_*oprD::lacZ* **(B)** were grown in LB medium. Samples were withdrawn at an OD_600_ of 2.0. The bars represent the β-galactosidase values conferred by the *opdP::lacZ* translational fusion gene encoded by plasmid pME6015P_tac_*opdP::lacZ* **(A)** and by the *oprD::lacZ* translational fusion gene encoded by plasmid pME6015P_tac_*oprD::lacZ* **(B)**. The error bars represent standard deviations from two and three independent experiments, respectively. **(C,D)** The strains PAO1Δ*crc*(pME4510) (purple bar) and PAO1Δ*crc*(pME4510*crc*_Flag_) (light purple bar) harboring either plasmid pME6015P_tac_ *opdP::lacZ* **(C)** or pME6015P_tac_*oprD::lacZ* **(D)** were grown in LB medium. Samples were withdrawn at an OD_600_ of 2.0. The bars represent the β-galactosidase values conferred by the *opdP::lacZ* translational fusion gene encoded by plasmid pME6015P_tac_*opdP::lacZ* **(C)** and by the *oprD::lacZ* translational fusion gene encoded by plasmid pME6015P_tac_*oprD::lacZ* **(D)**, respectively, in the presence or absence of ectopic *crc*_Flag_ expression. The error bars represent standard deviations from two independent experiments. Statistical analyses were performed in Excel with a two tailed distributed Student’s *T*-test of two sample arrays with unequal variance. ns *P* > 0.05, *P ≤ 0.05. Top panels, Crc_–__Flag_ and S1 levels in strains PAO1Δ*crc*(pME4510) (lane 1) and PAO1Δ*crc*(pME4510*crc*_Flag_) (lane 2) harboring either plasmid pME6015P_tac_*opdP::lacZ* **(C)** or pME6015P_tac_*oprD::lacZ* **(D)**. The Crc levels were determined by Western-blot analyses using anti-Crc antibodies. Immunodetection of ribosomal protein S1 served as a loading control.

Previous studies have shown that both, Hfq and Crc, are required for tight translational repression of mRNAs, which are subject to CCR (Sonnleitner and Bläsi, 2014; Moreno et al., 2015). Although the presence of Crc did not significantly enhance the affinity of Hfq for a RNA substrate, the simultaneous interactions of Crc with both binding partners resulted in an Hfq/Crc/RNA assembly with increased stability when compared with the Hfq/RNA complex alone (Sonnleitner et al., 2018). In other words, Crc can be regarded a co-repressor in Hfq/Crc repressive complexes. A recent structural study provided a rationale for the increased stability of the Hfq/Crc/RNA assembly by showing that the Hfq binding site on mRNA is sandwiched between both binding partners (Pei et al., 2019), which in turn can be readily reconciled with an increased translational repression observed for Hfq/Crc regulated genes in the presence of Crc (wild type strain) when compared to an isogenic *crc* deletion strain (Sonnleitner and Bläsi, 2014). Therefore, the following experiments were performed with the rationale that Crc should impact translation of genes that are assumed to be directly controlled by a Hfq/Crc repressive complex such as *opdP* rather than genes subject to canonical riboregulation such as *oprD*. Translation of *opdP* and *oprD* was monitored in strains PAO1 and PAO1Δ*crc* transformed with either plasmid pME6015P_tac_*opdP::lacZ* or pME6015P_tac_*oprD::lacZ*. As shown in Figure 4A, the β-galactosidase activity conferred by the *opdP::lacZ* translation was increased in the absence of Crc, i.e., the presence of Crc resulted in a stronger repression of *opdP::lacZ*. In contrast, the β-galactosidase activity conferred by the *oprD::lacZ* reporter gene was unaffected by Crc (Figure 4B). In line, ectopic expression of *crc-flag* resulted in repression of *opdP::lacZ* in strain PAO1Δ*crc*(pME6015P_tac_*opdP::lacZ*) (Figure 4C), whereas ectopic expression of *crc-flag* did not significantly change *oprD::lacZ* translation in strain PAO1Δ*crc*(pME6015P_tac_*oprD::lacZ*) (Figure 4D). These data strongly suggested that Crc contributes to Hfq-mediated direct translational regulation of *opdP*, i.e., that its translation is controlled by a Hfq/Crc repressive complex. As translational regulation of *oprD* was rather independent of Crc, these studies further support the notion that translational regulation of *oprD* is only subject to Hfq/EsrA/Sr0161-mediated riboregulation.

### High CrcZ Levels Increase *oprD* and *opdP* Translation

Transcription of the Hfq titrating RNA CrcZ is known to be induced by less preferred carbon sources such as mannitol (Sonnleitner et al., 2009). Therefore, we next tested whether the susceptibility toward imipenem is increased in an *oprD* deletion strain during growth in the presence of mannitol (high levels of CrcZ; Sonnleitner et al., 2009) when compared to the presence of succinate (low levels of CrcZ; Sonnleitner et al., 2009), i.e., whether translational repression of *opdP* by Hfq/Crc is relieved in the presence of CrcZ and in the absence of CCR. The disk diffusion assay revealed extended growth inhibition zones for the *oprD* mutant strain during growth in mannitol (Table 1), and therefore increased susceptibility to the tested antibiotic, consistent with the notion that *opdP* translation is controlled by Hfq/Crc. The same experiment conducted with an *opdP* deletion strain revealed a comparable result, indicating that CrcZ also interferes with Hfq/EsrA/Sr0161-mediated riboregulation of *oprD*. In support, ectopic overexpression of *crcZ* from plasmid pMMB*crcZ* in strains PAO1(pME6015P_tac_*oprD::lacZ*) (Supplementary Figure S4A) and PAO1(pME6015P_tac_*opdP::lacZ*) (Supplementary Figure S4B) confirmed that high levels of CrcZ increase both *oprD* and *opdP* translation. Again, these findings are in line with a reduced minimal inhibitory concentration (MIC) of imipenem upon ectopic overexpression of *crcZ* in PAO1 (Supplementary Figure S4C).

**TABLE 1:** Sensitivity toward imipenem during growth on different carbon-sources.

	Strains	PAO1	PAO1	PAO1 Δ*oprD*	PAO1 Δ*oprD*	PAO1 Δ*opdP*	PAO1 Δ*opdP*
	Media	BSM + 40 mM succinate	BSM + 40 mM mannitol	BSM + 40 mM succinate	BSM + 40 mM mannitol	BSM + 40 mM succinate	BSM + 40 mM mannitol
Antibiotic	Concentration	Inhibition zone [mm]^a^					
Imipenem	10 μg	22.25 ± 1.26	25.5 ± 1.29	18.5 ± 0.58	25.5 ± 0.58	20.5 ± 0.58	27.75 ± 2.06

*^*a*^Average of two independent experiments ± standard deviation.*

## Discussion

The outer membrane porins OprD and OpdP are required for the uptake of carbapenems (Tamber and Hancock, 2006). Here, we have verified and shown that Hfq is involved in negative translational riboregulation of *oprD* by the sRNAs EsrA and Sr0161. Moreover, we have provided evidence for Hfq/Crc-mediated regulation of *opdP*. Hence, the increased susceptibility of *P. aeruginosa hfq* deletion mutants toward imipenem (Pusic et al., 2018) can be rationalized at the molecular level by these studies. However, we cannot exclude that hitherto unknown Hfq-mediated regulatory circuits additionally impact *oprD* and *opdP* regulation.

**FIGURE 5:**
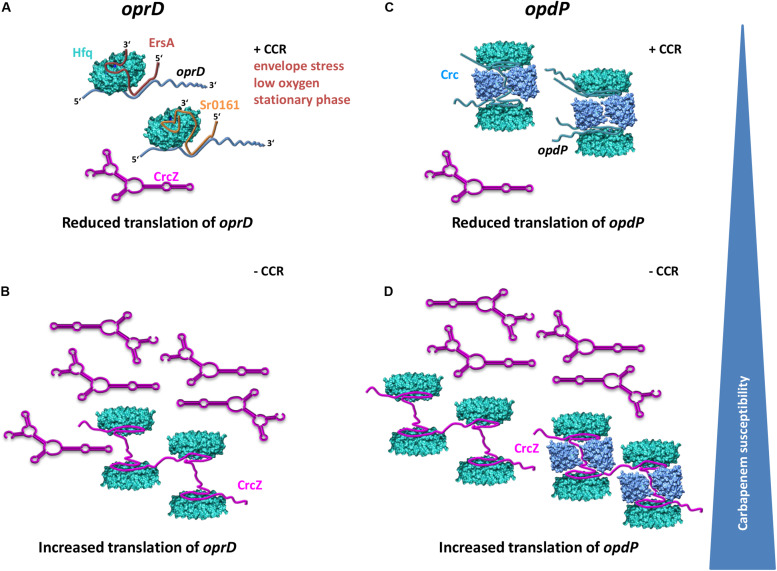
Model for distinctive translational regulation of *oprD* and *opdP* by Hfq/EsrA/Sr0161 and Hfq/Crc, respectively. **(A)** Under conditions of envelope stress, low oxygen and during stationary phase, EsrA is synthesized and represses translation of *oprD* in a Hfq dependent manner if the levels of the Hfq-titrating CrcZ RNA are low as it is the case during CCR (+CCR) (Sonnleitner et al., 2009). The environmental cues leading to synthesis of Sr0161, the Hfq-dependent second negative regulator of *oprD*, are unknown. **(B)** Alleviation of CCR results in increased levels of the RNA CrcZ, which will titrate Hfq and result in increased translation of *oprD*. **(C)** When CCR is in place, translation of *opdP* is reduced by a repressive Hfq/Crc complex. **(D)** Alleviation of CCR results in CrcZ synthesis and thus in titration of Hfq and/or Hfq/Crc complexes, which in turn will relieve translational repression of *oprD*. Translational repression of *oprD* by Hfq/ErsA/Sr0161 and of *opdP* by Hfq/Crc, respectively, will lead to reduced influx of carbapenems and thus increased resistance toward these antibiotics. In opposite, titration of Hfq and or Hfq/Crc by the RNA CrcZ will lead to increased translation of both, *oprD* and *opdP* concomitantly with an increased influx of and susceptibility to carbapenems.

Zhang et al. (2017) observed no specific amplicons of chimeras formed by Sr0161 and *oprD* mRNA in a Δ*hfq* strain. In addition, they showed that the *oprD* mRNA levels are increased in a Δ*hfq* strain. These experiments indicated that Sr0161-dependent negative regulation of *oprD* is Hfq dependent (Zhang et al., 2017). Using the triple mutant strain PAO1Δ*hfq*Δ*ersA*Δ*sr0161*(pME6015P_tac_*oprD::lacZ*), we have shown directly that translational repression of *oprD::lacZ* upon ectopic expression of either *esrA* or *sr0161* depends on Hfq (Figure 3D). Ectopic expression of Crc in strain PAO1Δ*crc*(pME6015P_tac_*oprD::lacZ*) insignificantly increased translation of *oprD::lacZ* (Figure 4D) rather than decreased it. This observation is consistent with our previous results in that over-expression of *crc* can interfere with Hfq-mediated riboregulation (Sonnleitner et al., 2018), and would argue against negative regulation of *oprD* translation by a repressive Hfq/Crc complex. In this context, it is also worth noting that the absence of Hfq in strain PAO1Δ*hfq*Δ*ersA*Δ*sr0161*(pME6015P_tac_*oprD::lacZ*) (Figure 3C) did not result in increased de-repression of *oprD::lacZ* translation when compared with strain PAO1Δ*ersA*Δ*sr0161*(pME6015P_tac_*oprD::lacZ*) (Figure 3C), again indicating that direct translational repression of *oprD* by Hfq/Crc does not occur in addition to Hfq/EsrA/Sr0161-mediated riboregulation (Figure 5A). This hypothesis also agrees with other studies (Corona et al., 2018), which indicated that *oprD* is not post-transcriptionally regulated by Crc.

Zhang et al. (2017) reported that deletion and over-expression of *sr0161* resulted in increased and decreased *opdP* mRNA levels, respectively, which was indicative for Sr0161-dependent regulation of *opdP*. However, our experiments performed with the translational *opdP::lacZ* reporter gene did not reveal direct evidence for an involvement of the sRNA Sr0161 in *opdP* translation (Figures 3A,B). Hence, we can only speculate that the observations made by Zhang et al. (2017) result from indirect effects. It is also worth noting that the putative Hfq and Sr0161 binding sites appear to overlap (Figure 2B). Thus, Hfq would be assumed to compete with sRNA binding rather than support the interaction with *opdP*. Our results are rather consistent with a model wherein a repressive Hfq/Crc complex prevents *opdP* translation when CCR is in place (Figure 5C). In support, our studies showed that *opdP::lacZ* translation is de-repressed and repressed in the absence of Crc and upon ectopic expression of *crc-flag*, respectively.

Hfq is titrated by the regulatory RNA CrcZ, which abrogates its function in Hfq-Crc-mediated translational repression during CCR (Sonnleitner and Bläsi, 2014) as well as in Hfq/sRNA-mediated riboregulation (Sonnleitner and Bläsi, 2014; Sonnleitner et al., 2017). Previous studies have shown that the levels of CrcZ are comparatively low when the cells are cultured in the presence of succinate (CCR) when compared with cells cultured in the presence of mannitol (no CCR) (Sonnleitner et al., 2009; Valentini et al., 2014). The model shown in Figure 5 therefore specifies that in the presence of low levels of CrcZ (succinate; CCR), the translation of both, *oprD* and *opdP*, is negatively regulated by Hfq/EsrA/Sr0161-mediated riboregulation (Figure 5A) and by Hfq/Crc repressive complexes (Figure 5C), respectively. In opposite, increasing levels of CrcZ (mannitol; no CCR) are anticipated to result in titration of Hfq, and in translation of *oprD and opdP* (Figures 5B,D). Hence, the differential imipenem susceptibility (Table 1) during growth on succinate and mannitol can thus be rationalized in light of Hfq-dependent *oprD*/*opdP* regulation and of the antagonizing function of the RNA CrcZ on Hfq regulatory complexes.

## Data Availability Statement

The datasets presented in this study can be found in online repositories. The names of the repository/repositories and accession number(s) can be found in the article/ Supplementary Material.

## Author Contributions

UB, ES, and PP conceived and designed the experiments. ES and PP performed the experiments. ES, PP, UB, and MW analyzed the data. ES and UB wrote the manuscript.

## Conflict of Interest

The authors declare that the research was conducted in the absence of any commercial or financial relationships that could be construed as a potential conflict of interest.

## Footnotes

1 https://www.viennabiocenter.org/facilities/

2 https://www.viennabiocenter.org/facilities/
