# Supplementary material for: Distinctive Regulation of Carbapenem Susceptibility in Pseudomonas aeruginosa by Hfq
Source: Front Microbiol. 2020 May 26;11:1001. doi: 10.3389/fmicb.2020.01001 (PMC7264166; doi:10.3389/fmicb.2020.01001)
Supplement: Supplementary file 1 [file Presentation_1.pdf]

Supplementary Material

Distinctive regulation of carbapenem susceptibility in *Pseudomonas aeruginosa* by Hfq

Elisabeth Sonnleitner*, Petra Pusic, Michael T. Wolfinger, and Udo Bläsi*

*** Correspondence:**

Elisabeth Sonnleitner: elisabeth.sonnleitner@univie.ac.at

Udo Bläsi: udo.blaesi@univie.ac

1 Supplementary Text

RNA-seq

Total RNA was prepared from two biological replicates of strains PAO1 and PAO1*hfq-*, respectively, after growth in LB medium to an OD_{600} of 1.0 and 3.0. 10 ml samples were withdrawn and total RNA was extracted using the hot phenol method (Leoni et al., 1996), contaminating DNA was removed by DNase (Roche) treatment followed by phenol-chloroform (pH 5.5) extraction and ethanol precipitation. To remove ribosomal RNAs, the Ribo-Zero™ Magnetic Kit for Gram-negative bacteria (Epicentre) was used according to the manufacturer's instructions. Libraries were constructed using NEBNext® Ultra™ Directional RNA Library Prep Kit from Illumina. 100 base pair single end sequence reads were generated using the Illumina HiSeq 2000 platform at the Vienna BioCenter Core Facility (https://www.viennabiocenter.org/facilities/). Sequencing adapter removal was performed with cutadapt (Martin, 2011). Mapping of the samples against the PAO1 reference genome (NCBI accession number NC_002516.2) was performed with Segemehl (Hofmann et al., 2009) with default parameters. Reads mapping to regions annotated as either rRNA or tRNA were discarded from all data and ignored for all follow up analysis steps. The mapped sequencing data were prepared for visualization using the ViennaNGS tool box, and visualized with the UCSC Genome Browser (Wolfinger et al., 2015). Reads per gene were counted using BEDTools (Quinlan and Hall, 2010) and the Refseq annotation of *P. aeruginosa* (NC_002516.2). Differential gene expression analysis was performed with DESeq (Anders and Huber, 2010). The raw sequencing data were deposited in the European nucleotide archive (ENA) as a study under the accession number PRJEB37368. Normalized total reads of *oprD* and *opdP*, their adjusted P-values and their fold change of the differential gene expression analysis are presented in **Supplementary Table S2**.

Northern blot analyses

Total RNA was purified using hot phenol (Leoni et al., 1996). The steady state levels of CrcZ and 5S rRNA (loading control) were determined by Northern-blotting using 4 µg of total RNA. The RNA samples were denatured for 5 min at 65°C in loading buffer containing 50% formamide, separated on a 8% polyacrylamide/8 M urea gel, and then transferred to a nylon membrane by electro-blotting. The RNA was cross-linked to the membrane by exposure to UV light. The membranes were hybridized with gene-specific ^{32}P-end-labelled oligonucleotides (CrcZ: K3 (5'-GCT GGA GTC GTT ACG TGT

TG-3’); 5S rRNA: I26 (5’-CCC CAC ACT ACC ATC GGC GAT GCG TCG-3’). The hybridization signals were visualized using a PhosphorImager (Molecular Dynamics).

Determination of the Minimal Inhibitory Concentration (MIC) by Evaluator Strips (E-test)

Bacterial cultures were grown in BSM medium supplemented with 40 mM succinate to an OD_{600} of 1.8. Then, 200 µl of cultures were plated on agar plates containing the respective media and MIC Evaluator strips for imipenem were applied with antibiotic concentrations of 0.002-32 µg/ml (Oxoid). The plates were incubated at 37°C and the MICs were determined by analyzing the growth inhibition zones. The MICs correspond to the lowest concentration of antibiotics that impeded growth.

Supplementary References

Anders, S., and Huber, W. (2010). Differential expression analysis for sequence count data. *Genome Biol* 11(10)**,** R106. doi: 10.1186/gb-2010-11-10-r106.

Fürste, J.P., Pansegrau, W., Frank, R., Blocker, H., Scholz, P., Bagdasarian, M., et al. (1986). Molecular cloning of the plasmid RP4 primase region in a multi-host-range *tac*P expression vector. *Gene* 48(1)**,** 119-131. doi: 10.1016/0378-1119(86)90358-6.

Han, K., Tjaden, B., and Lory, S. (2016). GRIL-seq provides a method for identifying direct targets of bacterial small regulatory RNA by *in vivo* proximity ligation. *Nat Microbiol* 2**,** 16239. doi: 10.1038/nmicrobiol.2016.239.

Heeb, S., Blumer, C., and Haas, D. (2002). Regulatory RNA as mediator in GacA/RsmA-dependent global control of exoproduct formation in *Pseudomonas fluorescens* CHA0. *J Bacteriol* 184(4)**,** 1046-1056. doi: 10.1128/jb.184.4.1046-1056.2002.

Holloway, B.W., Krishnapillai, V., and Morgan, A.F. (1979). Chromosomal genetics of *Pseudomonas*. *Microbiol Rev* 43(1)**,** 73-102.

Kulkarni, P.R., Jia, T., Kuehne, S.A., Kerkering, T.M., Morris, E.R., Searle, M.S., et al. (2014). A sequence-based approach for prediction of CsrA/RsmA targets in bacteria with experimental validation in *Pseudomonas aeruginosa*. *Nucleic Acids Res* 42(11)**,** 6811-6825. doi: 10.1093/nar/gku309.

Leoni, L., Ciervo, A., Orsi, N., and Visca, P. (1996). Iron-regulated transcription of the *pvdA* gene in *Pseudomonas aeruginosa*: effect of Fur and PvdS on promoter activity. *J Bacteriol* 178(8)**,** 2299-2313. doi: 10.1128/jb.178.8.2299-2313.1996.

Liberati, N.T., Urbach, J.M., Miyata, S., Lee, D.G., Drenkard, E., Wu, G., et al. (2006). An ordered, nonredundant library of *Pseudomonas aeruginosa* strain PA14 transposon insertion mutants. *Proc Natl Acad Sci U S A* 103(8)**,** 2833-2838. doi: 10.1073/pnas.0511100103.

Quinlan, A.R., and Hall, I.M. (2010). BEDTools: a flexible suite of utilities for comparing genomic features. *Bioinformatics* 26(6)**,** 841-842. doi: 10.1093/bioinformatics/btq033.

Rist, M., and Kertesz, M.A. (1998). Construction of improved plasmid vectors for promoter characterization in *Pseudomonas aeruginosa* and other gram-negative bacteria. *FEMS Microbiol Lett* 169(1)**,** 179-183. doi: 10.1111/j.1574-6968.1998.tb13315.x.

Schnider-Keel, U., Seematter, A., Maurhofer, M., Blumer, C., Duffy, B., Gigot-Bonnefoy, C., et al. (2000). Autoinduction of 2,4-diacetylphloroglucinol biosynthesis in the biocontrol agent *Pseudomonas fluorescens* CHA0 and repression by the bacterial metabolites salicylate and pyoluteorin. *J Bacteriol* 182(5)**,** 1215-1225. doi: 10.1128/jb.182.5.1215-1225.2000.

Wurtzel, O., Yoder-Himes, D.R., Han, K., Dandekar, A.A., Edelheit, S., Greenberg, E.P., et al. (2012). The single-nucleotide resolution transcriptome of *Pseudomonas aeruginosa* grown in body temperature. *PLoS Pathog* 8(9)**,** e1002945. doi: 10.1371/journal.ppat.1002945.

2 Supplementary Tables and Figures

2.1 Supplementary Tables

Supplementary Table S1. Strains and plasmids used in this study

Strain/plasmid	Genotype/relevant features	Source/reference
P. aeruginosa		
PAO1		(Holloway et al., 1979)
PAO1*hfq-*	*hfq::aadA*; Sp/Sm^R	(Sonnleitner et al., 2003)
PAO1Δ*hfq*	In frame deletion of *hfq*	(Sonnleitner et al., 2017)
PAOΔ*crc*	PAO6673, in frame deletion of *crc*	(Sonnleitner et al., 2009)
PAO1Δ*oprD*	In frame deletion of *occD1*/*oprD*	(Isabella et al., 2015)
PAO1Δ*opdP*	In frame deletion of *occD3*/*opdP*	(Isabella et al., 2015)
PAO1Δ*sr0161*	Deletion of *sr0161*	This study
PAO1Δ*ersA*Δ*sr0161*	Double deletion of *ersA* and *sr0161*	This study
PAO1Δ*hfq*Δ*ersA*Δ*sr0161*	Triple deletion of *hfq*, *ersA* and *sr0161*	This study
PAO1Δ*hfq*Δ*sr0161*	Double deletion of *hfq* and *sr0161*	This study
PA14		(Liberati et al., 2006)
PA14Δ*hfq*	In frame deletion of *hfq*	(Wurtzel et al., 2012)
**Plasmids**		
pME4510	Broad–host-range promoter-probe vector, Gm^R	(Rist and Kertesz, 1998)
pME4510hfq_{Flag}	pME4510 carrying PAO1 *hfq* fused to a Flag-tag encoding sequence under control of its authentic promoter	(Sonnleitner and Bläsi 2014)
pME4510crc_{Flag}	pME4510 carrying PAO1 *crc* fused to a Flag-tag encoding sequence under control of its authentic promoter	(Sonnleitner et al., 2018)
pKH6	pJN105 derived vector for L-arabinose (L-ara) inducible expression of sRNAs, Gm^R	(Han et al., 2016)
pKH6*ersA*	L-ara inducible expression of *ersA*	(Zhang et al., 2017)
pKH6*sr0161*	L-ara inducible expression of *sr0161*	(Zhang et al., 2017)
pME6032	$lacI^q$-P_{tac} expression vector, Tc^R	(Heeb et al., 2002)

pME6015	Cloning vector for translational *lacZ* fusions , Tc^R	(Schnider-Keel et al., 2000; Kulkarni et al., 2014)
pME6015P_{tac}	Cloning vector for translational *lacZ* fusions under the control of a P_{tac} promoter	This study
pME6015P_{tac}*oprD::lacZ*	pME6015P_{tac} containing a translational *oprD::lacZ* fusion	This study
pME6015P_{tac}*opdP::lacZ*	pME6015P_{tac} containing a translational *opdP::lacZ* fusion	This study
pEXG2	ColE1 suicide vector; *mob sacB* Gm^R	(Rietsch et al., 2005)
pEXG2-*sr0161*	pEXG2 with flanking regions to create an unmarked *sr0161* deletion	(Zhang et al., 2017)
pEXG2-*ersA*	pEXG2 with flanking regions to create an unmarked *ersA* deletion	(Zhang et al., 2017)
pMMB67HE	IncQ expression vector carrying an inducible P_{tac} promoter; Ap/Cb^R	(Fürste et al., 1986)
pMMB*crcZ*	pMMB67HE harboring *crcZ* under transcriptional control of P_{tac}	(Sonnleitner and Bläsi, 2014)

Supplementary Table S2 Transcript abundance of *oprD* and *opdP* in *P. aeruginosa* wild type and *hfq* deletion strains derived from different transcriptome analyses.

Comparison	Gene	Growth condition	Fold change (P_{adj}-value)	Base Mean wild type[a]	Base Mean mutant[a]	Reference
PA14Δ*hfq* vs PA14	*oprD*	SCFM, OD_{600}=2	5.0 (8.6 E-31)	10592	52700	Pusic et al., 2018
PA14Δ*hfq* vs PA14	*opdP*	SCFM, OD_{600}=2	20.7 (1.8 E-91)	443	9159	Pusic et al., 2018
PAO1*hfq*- vs PAO1	*oprD*	BSM complex, OD_{600}=1,5	2.3 (6.0 E-8)	18487	42194	Sonnleitner et al., 2018
PAO1*hfq*- vs PAO1	*opdP*	BSM complex, OD_{600}=1,5	22.6 (1.6 E-71)	107	2419	Sonnleitner et al., 2018
PAO1*hfq*- vs PAO1	*oprD*	LB, OD_{600}=1	2.6 (4.8 E-41)	75093	19629	This study
PAO1*hfq*- vs PAO1	*opdP*	LB, OD_{600}=1	11.6 (5.1 E-28)	1534	17803	This study
PAO1*hfq*- vs PAO1	*oprD*	LB, OD_{600}=3	3.9 (7.8 E-18)	14580	56468	This study
PAO1*hfq*- vs PAO1	*opdP*	LB, OD_{600}=3	13.9 (1.2 E-4)	177	2470	This study

[a] The results are given as base mean of two independent experiments

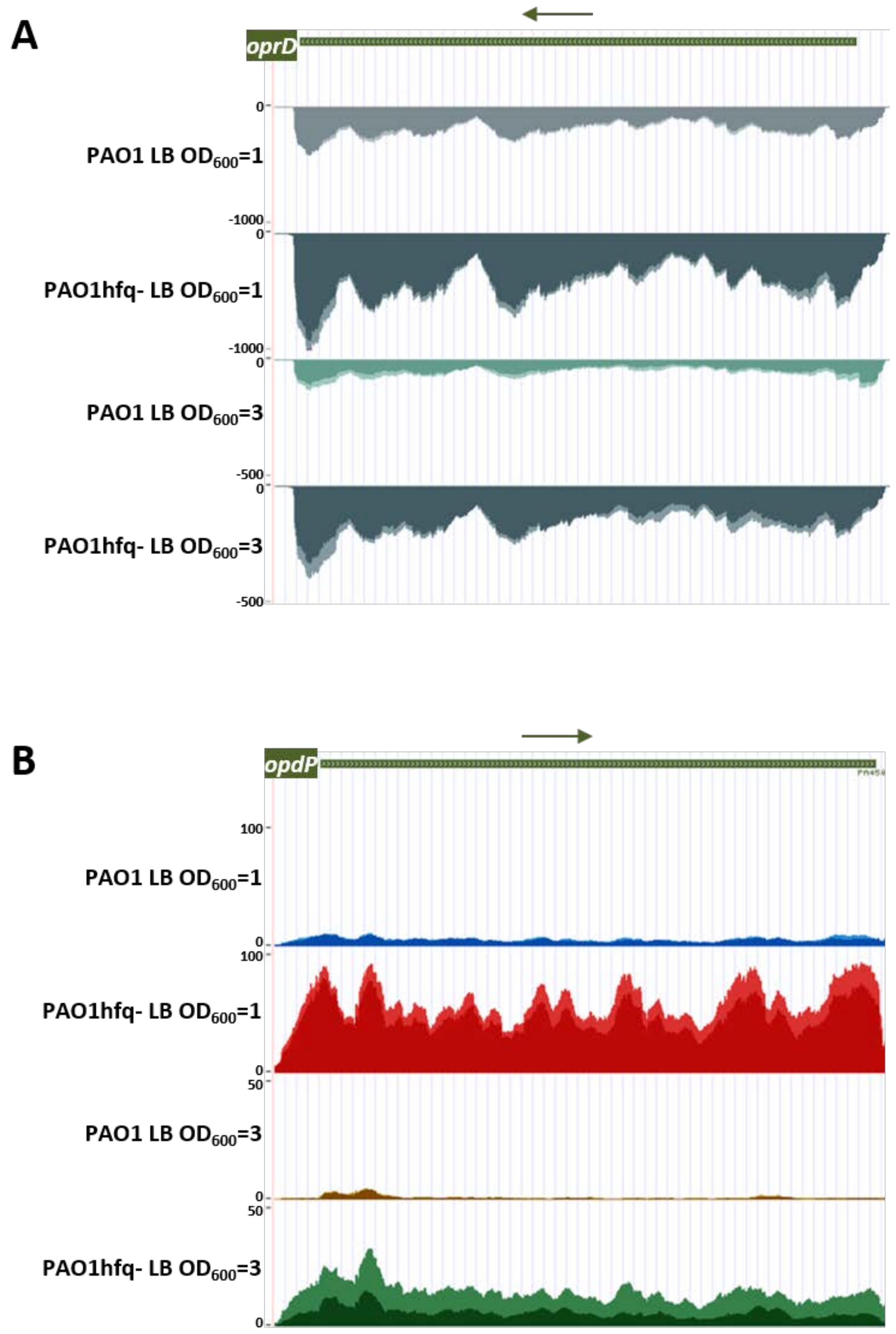

Supplementary Figure S1 Normalized read coverage visualized by the Genome Browser of the *oprD* **(A)** and *opdP* **(B)** transcripts after growth of PAO1 and PAO1*hfq*- in LB medium to an OD_{600} of 1.0 (upper panels) and 3.0 (lower panels), respectively. The arrows indicate transcriptional directionality.

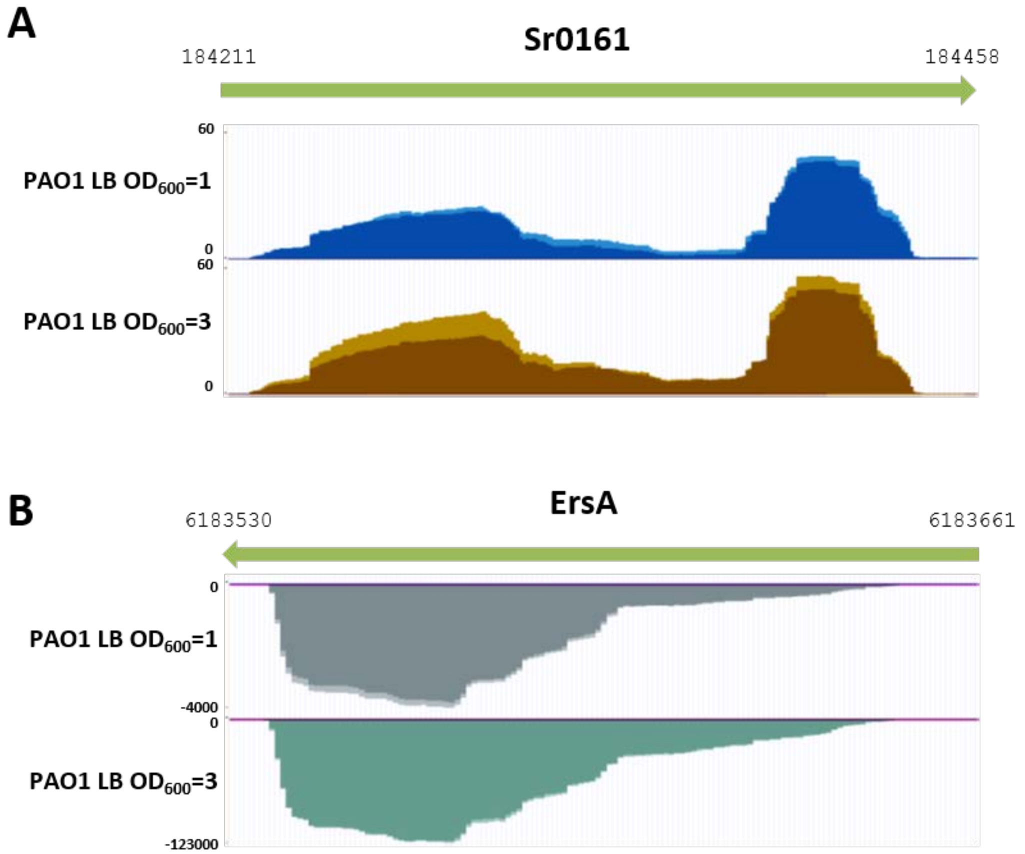

Supplementary Figure S2 Normalized read coverage visualized by the Genome Browser of the Sr0161 **(A)** and ErsA **(B)** sRNA transcripts after growth of PAO1 in LB medium to an OD_{600} of 1.0 (upper panels) and 3.0 (lower panels), respectively. The arrows indicate transcriptional directionality.

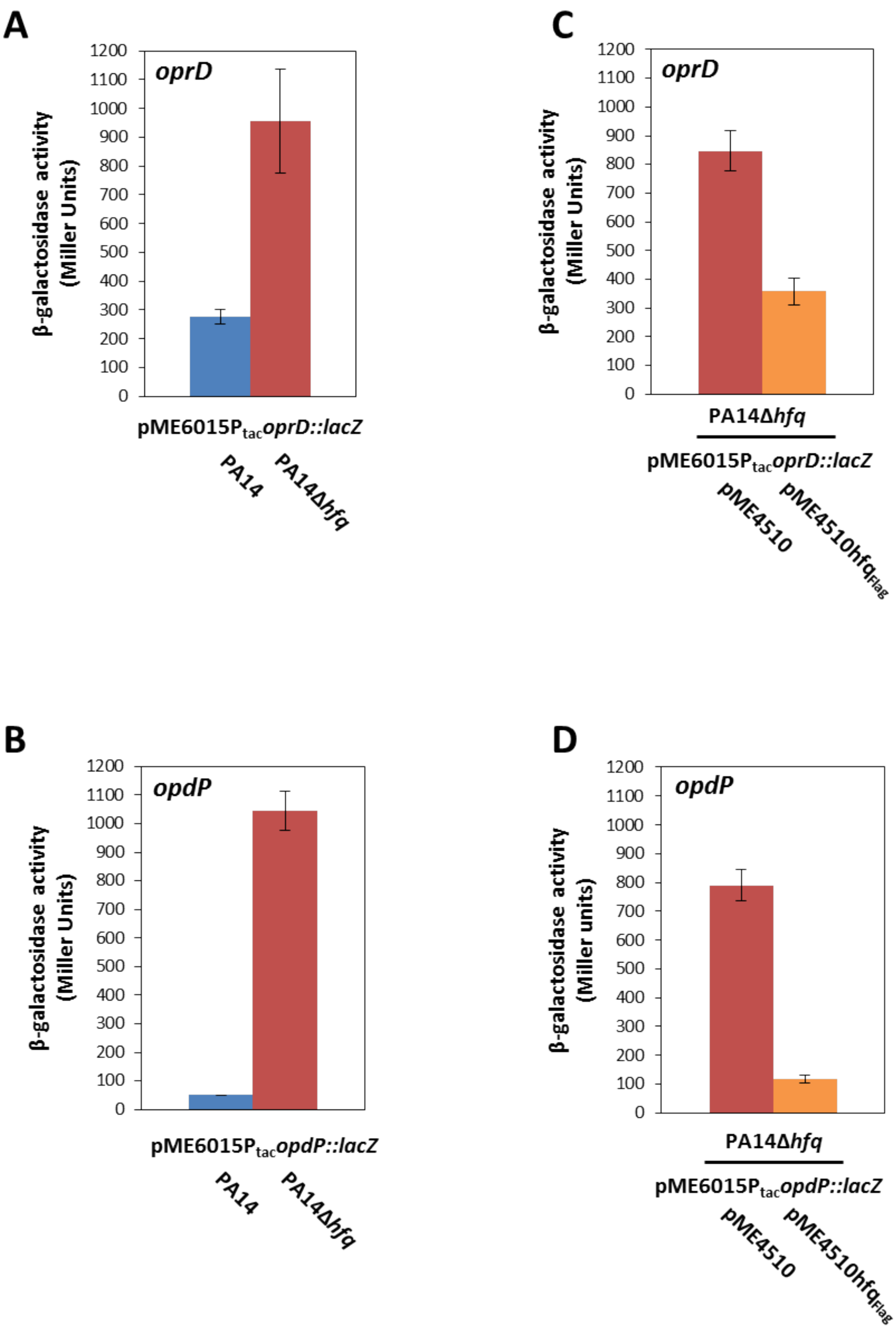

Supplementary Figure S3 Hfq negatively regulates *oprD* and *opdP* translation in strain PA14. (**A**, **B**) The strains PA14 (blue bar) and PA14Δ*hfq* (red bar) harboring either plasmid pME6015P$_{tac}$*oprD::lacZ* (**A**) or pME6015P$_{tac}$*opdP::lacZ* (**B**) were grown in synthetic cystic fibrosis medium (SCFM) (Palmer et al., 2007) containing 100 µM FeSO$_4$ (Tata et al., 2016). Samples were withdrawn at an OD$_{600}$ of 2.0.

The bars represent the β-galactosidase values conferred by the *oprD::lacZ* translational fusion gene encoded by plasmid pME6015P_{tac}*oprD::lacZ* (**A**) and by the *opdP::lacZ* translational fusion gene encoded by plasmid pME6015P_{tac}*opdP::lacZ* (**B**), respectively. The error bars represent standard deviations from two independent experiments. The strains PA14Δ*hfq*(pME4510) (red bar) and PA14Δ*hfq*(pME4510*hfq*$_{Flag}$) (orange bar) harboring either plasmid pME6015P_{tac}*oprD::lacZ* (**C**) or pME6015P_{tac}*opdP::lacZ* (**D**) were grown in SCFM medium containing 100 µM $FeSO_4$. Samples were withdrawn at an OD_{600} of 2.0. The bars represent the β-galactosidase values conferred by the *oprD::lacZ* translational fusion gene (**C**) and by the *opdP::lacZ* translational fusion gene (**D**), respectively, in the presence or absence of ectopic *hfq*$_{Flag}$ expression. The error bars represent standard deviations from three independent experiments.

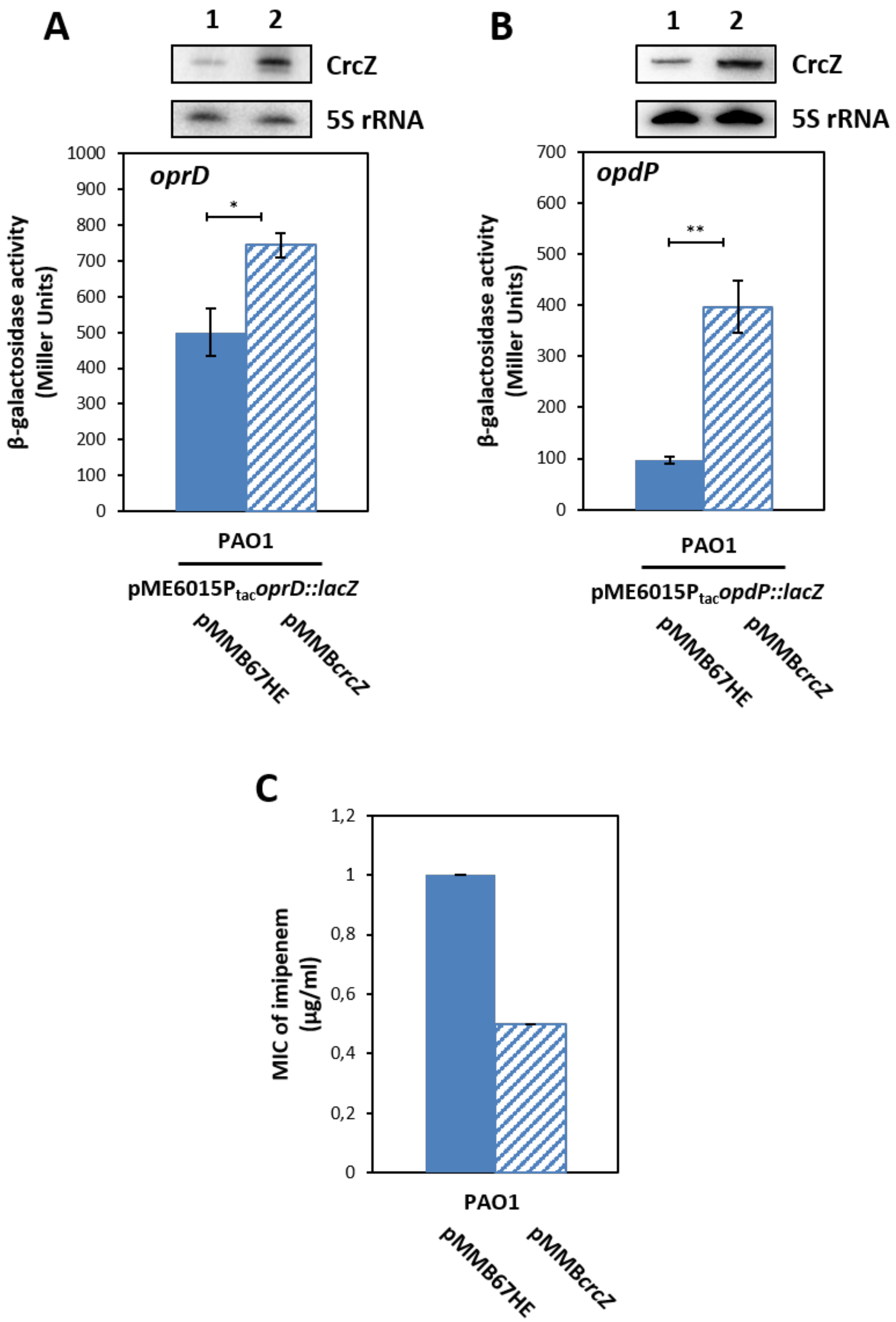

Supplementary Figure S4 Over-expression of *crcZ* results in increased *oprD*/*opdP* translation and susceptibility to imipenem. (**A**, **B**) The strains PAO1(pMMB67HE) (solid blue bar) and PAO1(pMMB*crcZ*) (striped blue bar) harboring either plasmid pME6015P$_{tac}$*oprD::lacZ* (**A**) or pME6015P$_{tac}$*opdP::lacZ* (**B**) were grown in BSM medium supplemented with 40 mM succinate. Samples were withdrawn at an OD$_{600}$ of 2.0. The bars represent the β-galactosidase values conferred

by the *oprD::lacZ* translational fusion gene encoded by plasmid pME6015P_{tac}*oprD::lacZ* (**A**) and by the *opdP::lacZ* translational fusion gene encoded by plasmid pME6015P_{tac}*opdP::lacZ* (**B**), respectively, in the presence and absence of ectopic *crcZ* expression. The error bars represent standard deviations from three independent experiments. Top panels, CrcZ and 5SrRNA levels in strain PAO1(pMMB67HE) (lane 1) and PAO1(pMMB*crcZ*) (lane 2) harboring either plasmid pME6015P_{tac}*oprD::lacZ* (**A**) or pME6015P_{tac}*opdP::lacZ* (**B**). CrcZ and 5S rRNA levels were determined by Northern-blot analyses as described in Supplementary Text S1. (**C**) Minimal inhibitory concentration (MIC) of imipenem in strain PAO1(pMMB67HE) (solid blue bar) and PAO1(pMMB*crcZ*) (striped blue bar) grown in BSM medium supplemented with 40 mM succinate. The MIC was determined by E-test strips (Oxoid) with an antibiotic concentration range from 32–0.002 µg/ml imipenem. The experiment was performed with two biological replicates.
